# Molecular mechanisms regulating lifespan and environmental stress responses

**DOI:** 10.1186/s41232-018-0080-y

**Published:** 2018-12-10

**Authors:** Saya Kishimoto, Masaharu Uno, Eisuke Nishida

**Affiliations:** 1 RIKEN Center for Biosystems Dynamics Research, 2-2-3 Minatojima Minamimachi, Chuo-ku, Kobe, 650-0047 Japan; 2 Department of Cell and Developmental Biology, Graduate School of Biostudies, Kyoto University, Sakyo-ku, Kyoto, 606-8502 Japan

**Keywords:** Aging, Lifespan extension, Environmental factor, Stress response, Epigenetics, Transgenerational inheritance

## Abstract

Throughout life, organisms are subjected to a variety of environmental perturbations, including temperature, nutrient conditions, and chemical agents. Exposure to external signals induces diverse changes in the physiological conditions of organisms. Genetically identical individuals exhibit highly phenotypic variations, which suggest that environmental variations among individuals can affect their phenotypes in a cumulative and inhomogeneous manner. The organismal phenotypes mediated by environmental conditions involve development, metabolic pathways, fertility, pathological processes, and even lifespan. It is clear that genetic factors influence the lifespan of organisms. Likewise, it is now increasingly recognized that environmental factors also have a large impact on the regulation of aging. Multiple studies have reported on the contribution of epigenetic signatures to the long-lasting phenotypic effects induced by environmental signals. Nevertheless, the mechanism of how environmental stimuli induce epigenetic changes at specific loci, which ultimately elicit phenotypic variations, is still largely unknown. Intriguingly, in some cases, the altered phenotypes associated with epigenetic changes could be stably passed on to the next generations. In this review, we discuss the environmental regulation of organismal viability, that is, longevity and stress resistance, and the relationship between this regulation and epigenetic factors, focusing on studies in the nematode *C. elegans*.

## Background

Aging is an inevitable event for most living organisms and is characterized by a progressive decline in physiological function. The aging process is strongly associated with the pathogenesis of many chronic diseases, including cardiovascular disorders, diabetes, cancer, and neurodegenerative diseases. Therefore, understanding the underlying molecular mechanisms of aging could be important for combating age-related diseases. In the 1980s, the isolation of the first long-lived strains of *Caenorhabditis elegans* established an emerging field of aging research [1]. A number of reports have since identified genetic factors and signaling pathways that are responsible for lifespan regulation [2]. Aging is currently regarded not only as just a passive process of physiological deterioration but also an actively controlled process that is conserved across species, from yeast to mammals. The well-conserved hallmarks of aging include the accumulation of genomic damages, epigenetic alterations, the loss of proteostasis, and deregulated nutrient sensing [3]. In fact, the aging process is affected by both genetic factors and environmental factors, and these factors are potently correlated with each other [4]. For example, environmental cues such as nutrient intake can interact with chromatin structures and alter transcriptional profiles, which could elicit stable changes in the aging of the organism. In this article, we review the current knowledge of aging research and highlight environmental stress responses that regulate organismal lifespan and stress resistance, with a focus on studies in *C. elegans*. We also discuss transgenerational effects of ancestral environmental challenges and their underlying molecular mechanisms.

## Main text

### Insulin/IGF-like signaling pathway in aging

**Fig. 1:**
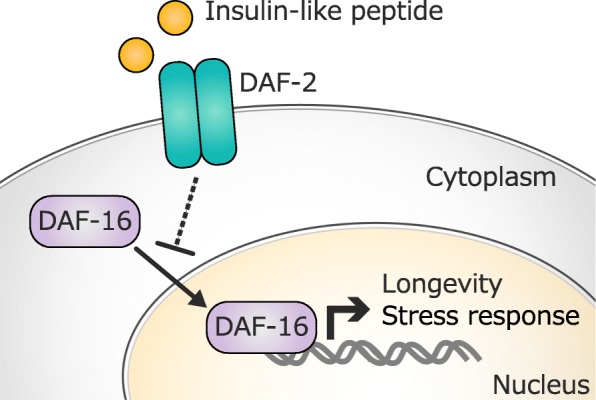
Schematic diagram of the IIS pathway-mediated longevity in *C. elegans*. Under reduced IIS, DAF-16 is translocated to the nucleus and activates the transcription of pro-longevity genes

In 1983, Klass reported the isolation of the first longevity mutants of *C. elegans* [1], and subsequently, one mutant was named *age-1* [5, 6]. The *age-1* gene encodes phosphatidylinositol 3-kinase (PI3K), which is a component of the insulin/insulin-like growth factor-1 signaling (IIS) pathway [5, 6]. The IIS pathway plays a pivotal role in the metabolism, growth, and lifespan by sensing nutrient levels. It was first identified as a lifespan-regulating signaling pathway in worms [7]. Many reports have demonstrated that the attenuation of the IIS pathway promotes lifespan extension and stress resistance. For instance, mutations that decrease the activity of *daf-2* (the *C. elegans* homolog of insulin/IGF receptor) more than double the lifespan of the animal [8]. Low IIS activity leads to the activation of the downstream transcription factor DAF-16 (the *C. elegans* homolog of FOXO), and DAF-16 upregulates a wide variety of genes, such as cellular stress response, antimicrobial and metabolic genes, which ultimately exerts pro-longevity effects (Fig. 1) [9–11]. Additionally, heat-shock transcription factor HSF-1 and the antioxidant-regulating transcription factor SKN-1 are also involved in the IIS-mediated lifespan regulation [12, 13]. The IIS pathway is highly conserved in a wide variety of species, and its suppression extends the lifespan in yeast, flies, and mice [14].

Additionally, TOR (target of rapamycin) signaling is a well-documented signaling pathway that regulates cell survival and growth, and, as in the case of the IIS pathway, the inhibition of TOR activity extends the lifespan of multiple organisms [15–18]. In *C. elegans*, the pro-longevity effect induced by low TOR signaling requires AAK-2 (a catalytic subunit of AMPK) and the transcription factor PHA-4/FOXA, which mediates autophagy (an intracellular proteolytic system crucially involved in lifespan regulation) [19, 20]. At least in *C. elegans*, TOR inhibition appears to increase the lifespan independently of DAF-16 [21, 22], suggesting that the TOR pathway may regulate longevity in a manner distinct from the IIS pathway. Recent studies have reported crosstalk between the TOR and IIS pathways for lifespan extension in some contexts [19, 23]; however, how they interact to modulate longevity in normal physiological conditions remains largely unclear.

### Dietary restriction-induced lifespan extension

Deregulated nutrient sensing is one of the hallmarks of aging [3]; in general, activating nutrient signaling results in the acceleration of aging. It has been consistently reported that dietary restriction (DR, a reduction in food intake without malnutrition) can reduce the nutrient signaling pathway and thereby increase the lifespan and health of many eukaryotes (including yeast, worms, flies, fish, and mice) [24]. DR can also protect against an age-related decline in function and pathologies in mammals. It also reduces the risk factors for cancer, diabetes, and cardiovascular diseases [24]. In addition, it has been reported that DR improves mitochondrial function via sirtuins, including SIRT1 and SIRT3, which contributes to lifespan extension [25]. Many other environmental factors (such as heat stress [26], oxidative stress [27, 28], and pheromones [29]) also induce phenotypic changes, which are relatively stable throughout life and can often prolong the organismal lifespan. Of these, DR is the most effective, well-documented intervention to extend the lifespan in many organisms. Several regimens of DR have been studied [30], including chronic calorie restriction, intermittent fasting, and depletion of specific nutrients such as amino acids. These methods extend the lifespan via distinct mechanisms that partially overlap. It is unlikely that a single pathway mediates the physiological outcomes of DR, as parallel and redundant pathways seem to contribute to the longevity induced by DR [24, 30, 31]. Both the IIS and the TOR signaling pathways sense the nutrient status of organisms. Therefore, the inhibition of these pathways is thought to mimic physiological conditions induced by food shortage. Consistently, genetic analysis has indicated that these pathways are involved in the DR-mediated longevity effects observed in worms and flies [24], although the relevant mechanisms may differ depending on the DR regimen used.

Intermittent fasting (IF) is one of the commonly used dietary restriction methods. In the IF regimen, animals are repeatedly subjected to periods of fasting. In *C. elegans*, Honjoh et al. demonstrated that IF (every 2 days) dramatically increased the lifespan (by approximately 60%) and delays age-related physiological declines [23]. The authors also found that IF-induced longevity is mediated through the TOR signaling pathway. RHEB-1 (an upstream activator of TOR) induces nuclear translocation of DAF-16, ultimately promoting the transcriptional activation of pro-longevity genes [23]. Another study revealed that, in IF-induced longevity, DAF-16 collaborates with the transcription factor AP-1 (consisting of JUN-1 and FOS-1), and KGB-1 (one of the *C. elegans* JUNK family members) activates AP-1 in response to fasting [32]. Additionally, SCF E3 ligase complexes are important transcriptional targets of these signaling pathways, and thereby IF induces enhanced protein ubiquitination [32], suggesting that protein homeostasis may contribute to IF-mediated longevity. Similarly, it is well-documented that autophagy, one of the major machineries that regulate protein homeostasis, plays a key role in various longevity pathways, including dietary restriction, in a diverse range of species [33, 34]. Clearance of cell damages by proteolytic systems appears to be important to extend lifespan and delay age-related diseases [35, 36].

### Epigenetic alterations associated with aging

Epigenetics is broadly defined as heritable changes in gene function without changes in the DNA coding sequences. The main mechanisms of epigenetic regulation involve DNA methylation, histone modifications, and non-coding RNAs. Epigenetic alterations are relatively stable throughout life and are linked to multiple biological processes, health, and diseases [37, 38]. Intriguingly, some epigenetic signatures have been reported to be biomarkers of aging [3, 39, 40]. For example, increases in histone H3 lysine 4 trimethylation (H3K4me3), H4K16ac, or H4K20me3 and decreases in H3K9me or H3K27me3 are known as age-associated epigenetic marks [39, 40]. The alteration of these marks is linked to changes in chromatin states around the marks, which may affect gene transcription levels and lead to subsequent biological outcomes. In addition, several studies have demonstrated that genetic manipulations of histone-modifying enzymes can influence the lifespan of multiple organisms. In *C. elegans*, inhibition of the H3K27me3 demethylase UTX-1 promotes longevity [41, 42]. Deficiency of components of the H3K4me3 methyltransferase complex (composed chiefly of SET-2, ASH-2, and WDR-5) increases lifespan [43]. Consistently, overexpression of the H3K4me3 demethylase RBR-2 extends lifespan, whereas suppression of RBR-2 shortens lifespan [43]. In *Drosophila melanogaster*, male flies with a deficiency of Lid (the fly ortholog of RBR-2) also show shortened lifespan [44]. However, it is not yet clear how changes in histone modifications regulate the aging process of organisms and whether the effects of histone modification on the regulation of the lifespan are evolutionarily conserved. Further studies are needed to better understand the role of epigenetic alterations in organismal aging. In addition to histone modifications, microRNAs (miRNAs, a class of small non-cording RNAs that post-transcriptionally regulate gene expression) are involved in epigenetic mechanisms, and some miRNAs regulate the lifespan of *C. elegans* under normal physiological conditions [45, 46]. Moreover, several studies have shown that long non-coding RNAs are implicated in longevity [47, 48]. It has also been reported that other epigenetic alterations, such as DNA methylation and chromatin remodeling, are also associated with aging [49–53].

**Fig. 2:**
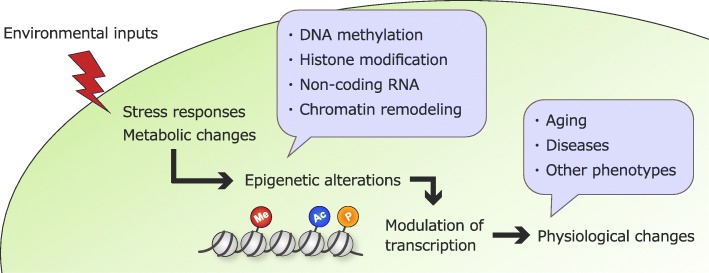
A possible model of epigenetic regulation in response to environmental factors. The white area represents environmental circumstances, and the green area represents the plausible responses of organisms to environmental stimuli. Epigenetic alterations (such as histone modification and chromatin remodeling) are known as the hallmarks of aging, and these changes are profoundly dictated by environmental stimuli [3, 4, 49]. Me, histone methylation; Ac, histone acetylation; P, histone phosphorylation

Epigenetic changes can be modulated by environmental signals. In fact, many metabolites generated by environmental factors, such as ATP and NAD^+^, often function as cofactors of epigenetic modifiers and substrates [54–57]. This suggests that there is a close relationship between environmental factor-modulated metabolism and epigenetic regulation. Consistent with this concept, epigenetic regulation is relevant to nutrient-sensing pathways, which directly affect metabolism. For instance, it has recently been suggested that MYS-1, the *C. elegans* homolog of the MYST family histone acetyltransferase Tip60, interacts with TRR-1 (one of PIKK family members) to regulate lifespan and stress resistance through the transcriptional upregulation of DAF-16, possibly mediated by histone acetylation that is catalyzed by MYS-1 [58]. The upregulation of DAF-16/FOXO mediated by the MYST complex was also shown in human cells [58], suggesting that there is an evolutionarily conserved role of histone acetylation. Additionally, a recent study showed that components of the miRNA machinery (including the miRNA-processing enzyme DRSH-1) are required for IF-induced longevity in *C. elegans* [59]. Moreover, it has been reported that certain miRNAs (*miR-228* and *miR-71*) mediate calorie restriction-induced longevity by interacting with the transcription factors PHA-4 and SKN-1 [60]. These findings suggest that epigenetic mechanisms are associated with the regulation of longevity and stress resistance in response to environmental stimuli. Therefore, epigenetic information may universally integrate environmental inputs throughout life and thus play an important role in the modulation of physiological phenomena, including aging (Fig. 2).

### Low-dose environmental stressors and longevity

**Fig. 3:**
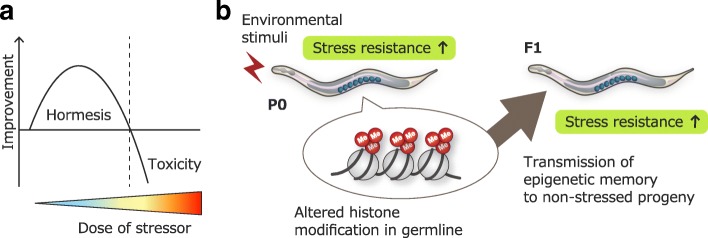
Transgenerational inheritance of acquired hormesis effects. **a** Bi-phasic dose-response curve. Low-dose treatment induces enhanced viability (hormesis effects), whereas exposure to high-dose stressors is detrimental. **b** Schematic model of heritable hormesis effects. Environmental stressors induce epigenetic alterations in the germline, which appear to be transmitted to the next generations and contribute to the viability of the offspring. Me, histone methylation

Multiple studies have demonstrated that exposure to low-dose environmental stressors elicits beneficial adaptive responses in organisms and increases their survivability, even though higher levels of stress exposure are detrimental [61–63]. The beneficial effects induced by non-lethal exposure to stressors have been called “hormesis” effects. Indeed, stress-induced hormesis effects can contribute to the lifespan extension and increase stress resistance. For instance, animals that undergo mild heat stress can acquire longevity and thermotolerance [64–66]. Such physiologically favorable outcomes of mild stress are considered to largely arise from improvements in the maintenance of cellular homeostasis, such as improved protein quality control. Hormesis effects have been reported across a diverse range of animal species [61]. Recently, it has been shown in *C. elegans* that exposure to low levels of environmental stressors during the developmental stages increases resistance to oxidative stress and proteotoxicity, suggesting the acquisition of hormesis effects [67]. Intriguingly, the authors found that the hormesis effects acquired in the parental generation could be transmitted to subsequent generations, with offspring showing enhanced stress resistance despite being raised under non-stressed conditions. The transgenerational inheritance continued up to the F3 generation. Moreover, the authors demonstrated that components of the histone H3K4me3 regulatory complex were required for the transgenerational inheritance of the acquired hormesis effects. In the parental generation, the H3K4me3 modifiers functioned in the germline and somehow communicated with DAF-16 and HSF-1 in the somatic tissues to induce and maintain epigenetic alterations. These epigenetic changes appear to be passed on to the next generations and contribute to eliciting hormesis effects for the survival of the offspring (Fig. 3). No direct evidence for the inheritance of stress-induced epigenetic alterations was shown in the study [67]. However, a growing number of studies support the involvement of epigenetic factors in transgenerational inheritance of various physiological changes (discussed below).

### Epigenetic regulation on transgenerational inheritance

Emerging evidence suggests that parental experiences can influence the phenotypes of their offspring in a diverse range of species, including mammals [68, 69]. This includes “intergenerational” inheritance, which often results from maternal effects or direct exposure of the offspring to environmental cues in utero. However, exposure of only parental males to stimuli has also been shown to induce phenotypic variations in their progeny [70, 71], and these heritable effects could last several generations. These findings suggest that transgenerational inheritance is mediated by invertible and non-genetic mechanisms, presumably epigenetic mechanisms [72–75]. In *Drosophila*, heat shock-induced heterochromatin disruption was transmitted over multiple generations, presenting as a phenotypic change, but the chromatin state eventually returned to normal [76]. In mice, learning associated with the olfactory system resulted in behavioral and neuroanatomical changes in the descendant generations, which were accompanied by epigenetic alterations involving the olfactory receptor gene [77]. Most examples of transgenerational inheritance are either neutral or harmful to organisms. In some cases, however, beneficial effects induced by parental experiences can be transmitted to the next generations (including the hormesis effect mentioned above [67]). Such heritable phenotypic changes are thought to be an adaptive response that ensures the survival of offspring in harsh environmental conditions.

In the past decade, research in the field has focused on the molecular insights into a non-Mendelian mode of inheritance and has provided some plausible epigenetic mechanisms. In general, reprogramming of the germline removes epigenetic signatures imposed by the environment in parental generations so that the offspring develops properly, according to the appropriate gene regulation. However, epigenetic alterations can sometimes be retained and passed on to the next generation [78]. Recent data in *C. elegans* provided evidence for the transmission of parental histone modification patterns to embryos [79]. Additionally, many studies have demonstrated that small non-coding RNAs (including miRNA, small interfering RNA (siRNA), and Piwi-interacting RNA (piRNA)) are involved in transgenerational epigenetic inheritance [80, 81]. For example, in *C. elegans*, starvation in the parental generation alters their small RNA expression profiles, which are maintained and contribute to the longevity of their offspring for multiple generations [82]. Collectively, histone modifications and small RNAs are thought to play a pivotal role in transgenerational inheritance by maintaining ancestral epigenetic memories.

## Conclusions

Organismal lifespan is regulated by both genetic and environmental factors. Genetic mutations (including those in the IIS and TOR pathways) can induce longevity, and environmental stimuli (such as nutrient) also change the aging process. Dietary restriction, one such environmental factor, can effectively extend the lifespan in a diverse range of species. Several factors in the evolutionarily conserved longevity pathways are thought to modulate the epigenetic states of organisms in response to environmental changes and thereby alter their lifespan and stress resistance. In fact, phenotypic changes via epigenetic alterations can continue not only throughout life but also through subsequent generations. Long-lasting epigenetic perturbation seems to be associated with age-related diseases, including cancer and psychiatric disorders, and thus may influence the health and disease state of the offspring [83]. Given the plasticity of epigenetic states, epigenetic modifiers could be potential therapeutic targets. A better understanding of the mechanisms of epigenetic regulation in response to environmental signals may help delay age-related diseases and extend healthy lifespan.

## Abbreviations

DR: Dietary restriction
IF: Intermittent fasting
IIS: Insulin/insulin-like growth factor-1 signaling
miRNA: MicroRNA
TOR: Target of rapamycin
